# Prevalence of post kala-azar dermal leishmaniasis (PKDL) and treatment seeking behavior of PKDL patients in Nepal

**DOI:** 10.1371/journal.pntd.0011138

**Published:** 2023-02-09

**Authors:** Anand Ballabh Joshi, Megha Raj Banjara, Murari Lal Das, Pragyan Ghale, Krishna Raj Pant, Niraj Parajuli, Uttam Raj Pyakurel, Gokarna Raj Dahal, Chuman Lal Das, Axel Kroeger, Abraham Aseffa

**Affiliations:** 1 Public Health and Infectious Disease Research Center (PHIDReC), Kathmandu, Nepal; 2 Central Department of Microbiology, Tribhuvan University, Kirtipur, Kathmandu, Nepal; 3 WHO Special Programme for Research and Training in Tropical Diseases (WHO/TDR), Geneva, Switzerland; 4 Bir Hospital, Kathmandu, Nepal; 5 Epidemiology and Disease Control Division, Department of Health Services, Teku, Kathmandu, Nepal; 6 Centre for Medicine and Society, Albert-Ludwigs-University, Freiburg, Germany; Institute of Postgraduate Medical Education and Research, INDIA

## Abstract

**Background:**

In Nepal, the burden of post kala-azar dermal leishmaniasis (PKDL) is not known since there is no active case detection of PKDL by the national programme. PKDL patients could pose a challenge to sustain visceral leishmaniasis (VL) elimination. The objective of this study was to determine the prevalence of PKDL and assess PKDL patients’ knowledge on VL and PKDL, and stigma associated with PKDL.

**Methodology/Principal findings:**

Household surveys were conducted in 98 VL endemic villages of five districts that reported the highest number of VL cases within 2018–2021. A total of 6,821 households with 40373 individuals were screened for PKDL. Cases with skin lesions were referred to hospitals and examined by dermatologists. Suspected PKDL cases were tested with rK39 and smear microscopy from skin lesions. An integrated diagnostic approach was implemented in two hospitals with a focus on management of leprosy cases where cases with non-leprosy skin lesions were tested for PKDL with rK39. Confirmed PKDL patients were interviewed to assess knowledge and stigma associated with PKDL, using explanatory model interview catalogue (EMIC) with maximum score of 36. Among 147 cases with skin lesions in the survey, 9 (6.12%) were confirmed as PKDL by dermatologists at the hospital. The prevalence of PKDL was 2.23 per 10,000 population. Among these 9 PKDL cases, 5 had a past history of VL and 4 did not. PKDL cases without a past history of VL were detected among the “new foci”, Surkhet but none in Palpa. None of the cases negative for leprosy were positive for PKDL. There was very limited knowledge of PKDL and VL among PKDL cases. PKDL patients suffered to some degree from social and psychological stigma (mean ± s.d. score = 17.89 ± 12.84).

**Conclusions/Significance:**

Strengthening the programme in PKDL case detection and management would probably contribute to sustenance of VL elimination. Awareness raising activities to promote knowledge and reduce social stigma should be conducted in VL endemic areas.

## Author summary

Post kala-azar dermal leishmaniasis (PKDL) is a sequel of visceral leishmaniasis (VL) and occurs in VL endemic areas. PKDL patients can be a reservoir for *Leishmania donovani* parasites and thus sustaining the occurrence of VL. Since Bangladesh, India and Nepal have targeted the elimination of VL, it is important to eliminate PKDL as well. However, in Nepal there is no active case detection of PKDL and the real burden of PKDL is not known. This community based survey encountered a PKDL prevalence of 2.23 per 10,000 population. PKDL patients had limited knowledge of VL and PKDL. They suffered to some degree from social and psychological stigma. Therefore, PKDL case detection and management should be strengthened in the national programme in the context of the VL elimination initiative.

## Introduction

Visceral leishmaniasis (VL) is a public health problem in Nepal and it has been targeted for elimination. The new cases of VL in Nepal in the years 2019, 2020 and 2021 were 216, 186 and 212 respectively [1].

Among the different manifestations of leishmaniasis, post kala-azar dermal leishmaniasis (PKDL) occurs in areas endemic for *Leishmania donovani*. PKDL is a sequelae of VL characterized by hypo-pigmented macular, popular, nodular or polymorphic skin lesions. These skin lesions are often confounded with other skin conditions such as leprosy or vitiligo causing difficultly in the differential diagnosis. PKDL is a chronic condition where patients may serve as reservoirs for VL parasites facilitating further transmission of VL in communities. Diagnosis and treatment of PKDL is therefore an important component of the VL elimination effort.

The risk factors for PKDL are not fully understood. A study conducted in Bangladesh through active case search for VL and PKDL cases showed that among 22,699 individuals surveyed, 813 (3.6%) had VL in the past and 79 (0.35%) had PKDL with eight additional PKDL patients without history of VL during 2002–2008 [2]. Further, a cohort study during 2014–2018 in Bangladesh revealed that the development of PKDL and VL relapses (VLRs) is related to treatment regimens for VL. Sodium stibogluconate (SSG) and multi-dose liposomal amphotericin B (L-AmB) resulted in less incidence of PKDL and VLRs compared to other treatment regimens [3]. Follow up of VL patients treated with single dose AmBisome, AmBisome-miltefosine, and miltefosine-paromomycin in India between 2012–2014 revealed a PKDL incidence density of 1.29, 1.45 and 2.65 per 1000 person months respectively. Children <12 years of age and females were high risk groups for PKDL [4]. A retrospective study conducted in 2010 in Nepal showed that among VL patients treated with sodium stibogluconate (SSG) between 2000–2009, 2.4% had PKDL [5].

It is difficult to predict who will develop PKDL. Poor treatment compliance, young age (5–17 years), malnutrition, co-infection with HIV and treatment of HIV as well as long time environmental arsenic exposure may all be associated with the development of PKDL [6].

A recent xenodiagnosis study revealed that nodular and macular PKDL patients are infectious to sandflies [7]. PKDL is thus recognized as a threat to the VL elimination effort, and the development of strategies for case finding, diagnosis, and treatment is one of the priority objectives of the Kala-azar Elimination Programme. However, the true burden of PKDL in Nepal is unknown. There is no active case search for PKDL in the national programme. Passive case detection always underestimates PKDL and an active case search in the community is a better way to determine the real burden of PKDL. Active PKDL case detection and prompt treatment should be an integral part of VL elimination programs. How different VL treatment regimens affect the risk of PKDL needs to be clarified. It is essential to detect PKDL cases in the community and ensure their complete treatment, determine the prevalence of PKDL and assess the relationship of PKDL to the VL treatment regimens applied.

PKDL patients do not always feel sick and they may not seek treatment. Such inappropriate treatment seeking behavior of PKDL patients may allow the disease to stay chronic, increase its severity and enhance its transmissibility. Lack of knowledge and perceived stigma may cause patient delay in seeking treatment. This study was conducted to determine the prevalence of PKDL and identify the health care seeking behavior of and attached stigma to PKDL patients in Nepal.

## Methods

### Ethics statement

PKDL case screening was conducted in the community. Blood samples were collected from suspected PKDL patients with skin lesions and rK39 tests were performed after obtaining written informed consent and assent where applicable. PKDL patients after confirmatory test in hospitals and recorded past PKDL cases were interviewed for their treatment seeking behavior. The written consent or assent was obtained in people’s house where the active case detection of PKDL was conducted. The formal written consent was obtained from the parent/guardian in case of child participant. The consent or assent was obtained by a trained research assistant before screening interview in the house and examination of skin lesions. In hospitals with a focus on management of leprosy cases, the consent was obtained by laboratory technicians. Those who agreed were tested for PKDL using rK39 tests. Ethical approval was obtained from World Health Organization-Ethics Review Committee (Ref. No.: ERC 0003532) and the Nepal Health Research Council (Ref. No.: 3084).

### Study design

This implementation research was conducted to determine the prevalence of PKDL through active case search in VL endemic districts/communities. Surveys were conducted to screen PKDL cases in 98 VL endemic villages of five districts that reported high numbers of VL cases within the last four years (2018–2021). The detection of PKDL using the integrated diagnostic approach was also implemented in two hospitals with a focus on management of leprosy cases where cases with skin lesions negative for leprosy were tested with rK39 test to detect PKDL.

### Study sites and population

The number of VL cases reported in five high VL endemic districts by village within the last four years (2018–2021) was obtained from the Epidemiology and Disease Control Division of the Ministry of Health and Population. The total number of VL cases reported in Morang, Siraha, Sarlahi, Palpa and Surkhet within 2018–2021 were 12, 13, 15, 25 and 52 respectively. A total of 40,373 residents of 98 villages (26 from Morang, 20 from Palpa, 10 from Sarlahi, 12 from Siraha, and 30 from Surkhet) were included. Additionally, clinicians of two hospitals with a focus on management of leprosy cases in the VL/PKDL endemic areas were trained on the differential diagnoses of PKDL in patients with skin lesions who were negative for leprosy.

### Sample size and sampling

A total of 98 VL endemic villages of five districts that reported high numbers of VL cases within the last four years were selected and all inhabitants were surveyed for skin lesions for detection of PKDL. In this survey, 40,373 people were enumerated and screened in the villages by the research team. Nine clinicians and 14 lab technicians involved in the diagnosis and treatment of leprosy in two leprosy hospitals located in the VL/PKDL endemic areas were trained on detection of PKDL.

### Co-ordination with national VL programme

The PKDL survey as well as the integrated approach of PKDL diagnosis in leprosy care facilities was conducted in coordination with the Epidemiology and Disease Control Division (EDCD) of the Ministry of Health and Population of Nepal.

Active case detection of PKDL in the community and at the leprosy hospitals, interviews of PKDL cases, and the training of clinicians and laboratory technicians on clinical presentation of PKDL case and laboratory diagnosis were conducted at leprosy hospitals by the research team as part of the research activities. Programme staff from national, provincial and district levels participated in the survey of PKDL and in the training of clinicians in leprosy hospitals on PKDL diagnosis.

### Survey of PKDL

Rapport was built early with the communities informing them about the study. The research information was disseminated to the communities by Female Community Health Volunteers (FCHVs) and local leaders of rural and urban municipalities. They informed the communities about the screening program to be implemented by Public Health and Infectious Disease Research Center (PHIDReC) in collaboration with the District Health Office and Epidemiology and Disease Control Division, Ministry of Health and Population.

A total of 6,821 households was visited by the research assistants including FCHVs of the respective villages to screen for PKDL cases. After obtaining informed consent, demographic and clinical data were collected using a structured questionnaire to ascertain past and current cases of VL and PKDL. Data included month and year of VL and PKDL symptom onset and treatment. Any cases with suspicious skin lesions were referred to the hospitals and examined at Koshi Hospital in Morang, Siraha Hospital in Siraha, Sarlahi Hospital in Sarlahi, United Mission Hospital in Palpa and Karnali Province Hospital in Surkhet by consultant physicians and dermatologists. Suspected PKDL cases with skin lesions were tested with rK39 RDT and microscopy of lesion smear in the hospitals. The research team arranged the transport facility for suspected PKDL and leprosy cases to the hospitals. All previously diagnosed cases were included in the survey. The clinicians and laboratory technicians working in the hospitals with a focus on management of leprosy cases were trained by a VL and leprosy treating physician from Bir Hospital, Kathmandu. rK39 test was performed in suspected PKDL cases negative for leprosy in hospitals with a focus on management of leprosy cases. All study team members received adequate training on protective measures against COVID-19 and exercised recommended protective measures against COVID-19 infection.

### Interview of PKDL cases

PKDL cases actively detected through the survey were interviewed using a semi-structured questionnaire on their treatment seeking behavior, care of skin lesions, attitude of the family and delay time for seeking care, treatment of VL in the past, any other cases of VL and PKDL in the household, and compliance with anti-leishmanial drug treatment.

Twelve items on a stigma scale from the explanatory model interview catalogue (EMIC) was used to assess the perceived stigma [8]. This scale contained 12 items and each question had four options ‘no’ ‘uncertain’ ‘possibly’ ‘yes’ and all questions are scored from 0 to 3. Maximum obtainable score is 36 and minimum is 0. The higher the score of EMIC, the higher the perceived stigma.

### Data management and analysis

Data was collected with the Kobo toolbox, a survey app from UNOCHA (https://kobo.humanitarianresponse.info). Data was imported into excel and cleaned. Further data cleaning and data analysis was performed on IBM SPSS Statistics 21. Descriptive statistics were generated and presented to describe interview findings, skin lesion cases, PKDL suspects and confirmed cases with clinical and laboratory examination outcome, knowledge and stigma related findings and other characteristics of study participants.

## Results

### Background characteristics of screened population for PKDL

A total of 40373 individuals from 6821 households of 98 villages in the five chosen districts were screened. Of these participants 51.9% were male and 48.1% female. The highest numbers of individuals were screened in Sarlahi and the least in Palpa. Among the screened participants, 41.8% were younger than 20 years of age whereas participants older than 60 years made up less than 14% (Table 1).

**Table 1:** Characteristics of screened population in five districts.

Districts	Morang No.(%)	Palpa No.(%)	Sarlahi No.(%)	Siraha No.(%)	Surkhet No.(%)	Total No.(%)
Villages	26(26.5)	20(20.4)	10(10.2)	12(12.2)	30(30.6)	98(100)
Screened population	9941(24.6)	1027(2.5)	15800(39.1)	10139(25.1)	3466(8.6)	40373(100)
Male	5092(51.2)	519(50.5)	8258(52.3)	5347(52.7)	1739(50.2)	20955(51.9)
Female	4849(48.8)	508(49.5)	7542(47.7)	4792(47.3)	1727(49.8)	19418(48.1)
Age group (Years)						
≤10	1999	147	3611	2088	598	8443(20.9)
11–20	1973	189	3478	2034	767	8441(20.9)
21–30	2061	208	3082	2002	735	8088(20)
31–40	1429	180	2064	1547	563	5783(14.3)
41–50	982	105	1522	973	378	3960(9.8)
51–60	741	81	971	687	179	2659(6.5)
61–70	575	79	696	592	161	2103(5.2)
≥71	181	38	376	216	85	896(2.2)

### Determination of prevalence of PKDL

Forty-seven referred patients by the research team and 26 suspected PKDL cases were tested in Morang. Fifty eight were referred and 48 individuals were taken to the hospital to check for PKDL in Siraha. Eight were referred and all 8 individuals were tested for PKDL in Palpa. Ten were referred and all 10 suspected cases were tested in Sarlahi. Twenty-five were referred and 23 were tested for PKDL in Surkhet.

There were a total of 151 individuals amongst the population with a past history of VL. Among 147 individuals with PKDL like skin lesions, 21 had a past history of VL. Nine of the 147 individuals were diagnosed with PKDL. Five of these had a past history of VL and 4 did not (Table 2). The past VL cases among the screened population stated different periods of occurrence ranging up to 20 years. Among PKDL cases, 5 had a previous history of VL within the last 4 years.

**Table 2:** Individuals with VL in the past and PKDL like skin lesions.

District	Individuals with past history of VL No.(%)	PKDL like lesions with past history of VL No.(%)	Confirmed PKDL with past history of VL No.(%)	PKDL like lesions without past history of VL No.(%)	Confirmed PKDL without past history of VL	PKDL cases
Morang	57(37.7)	10(47.6)	4(80)	36(28.5)	0	4
Palpa	15(9.9)	6(28.6)	0(0)	2(1.6)	0	0
Sarlahi	35(23.2)	0(0)	0(0)	10(7.9)	0	0
Siraha	26(17.2)	2(9.5)	1(20)	56(44.4)	0	1
Surkhet	18(11.9)	3(14.2)	0(0)	22(17.5)	4	4
Total	**151**	**21**	**5**	**126**	**4**	**9**

The prevalence of PKDL per 10,000 population was calculated to be 2.23. Among screened cases with a past history of VL, five (3.31%) had PKDL. Four (0.01%) PKDL cases did not have any past history of VL. Among 147 individuals with skin lesions, nine (6.12%) were diagnosed as PKDL. None of the individuals with skin lesions was positive for leprosy. The skin lesion cases without PKDL had fungal infections and other skin conditions (Table 3).

**Table 3:** Prevalence of PKDL based on different characteristics of screened individuals.

Particulars	Values
Prevalence of PKDL per 10,000 population	9/40373(2.23)
Prevalence of PKDL amongst individuals with past history of VL	5/151(3.31%)
Prevalence of PKDL amongst individuals without past history of VL	4/40222(0.01%)
Prevalence of PKDL amongst skin lesion cases	9/147(6.12%)

Eighty (54.4%) of the screened individuals with skin lesions had macular lesions while 31 (21.5%) had papular lesions, 20 (13.9%) had nodular and 18 (12.5%) individuals had polymorphic skin lesions

Among the 151 cases with a history of past VL, the majority reported that they were diagnosed and treated at public hospitals. A few cases reported that they were diagnosed and treated at private hospitals in India. Three of the past VL cases reported that they had not completed their treatment. One of them had received an ayurvedic treatment at home. The second one with papular skin lesions was referred to the closest government hospital and the third stopped treatment because of unavailability of the drug; then he developed macular skin lesions and was tested positive for PKDL.

### Risk factors associated with PKDL including VL treatment regimens

Among the 151 individuals with a past history of VL, 52 individuals answered the question on the drug used for their treatment. Fourteen out of the 52 individuals reported that they used more than one drug for their treatment. Not all the patients with a history of kala-azar had information on the type of medicine used for their treatment. However, from the data available, miltefosine (28, 53.8%) was found to be the most common drug used for treatment. Paromomycin (13, 25%) was the second most common, followed by multiple doses of L-AMB (8, 15.4%). A single dose of L-AMB (5, 9.6%), sodium stibogluconate (4, 7.7%) and amphotericin B (4, 7.7%) were rarely prescribed. Amongst the patients positive for PKDL with a past history of kala-azar (n = 5), 1 had received miltefosine, 2 had received multiple doses of L-AmB, 1 had received a combination drugs, and one did not know the drug used for VL treatment.

Among 9 PKDL cases, 4 were from Morang and Surkhet each and 1 from Siraha. The four cases from Morang and Surkhet were reported to come from the same municipality. In Morang, 3 of the cases were from the same village Babiyabirta and 1 was from Dohmana. In the case of Surkhet all 4 cases were from different villages, 1 from Narayantol, 1 from Sathkhola, 1 from Mulpani and 1 from Bheriganga.

The age distribution of the PKDL cases showed that 5 (55.5%) were in the age group of 20–45 years, 2 (22.2%) were less than 20 years old, 1 (11.1%) was in the 46–60 age group and the remaining 1 (11.1%) was above 60 years of age. Amongst them 4 were male and 5 were female. Four of them had secondary education, 2 of them had no formal education, 1 was literate and 2 had primary education. Two of them were farmers, 2 were students, 1 was a house wife, 1 labor, 1 worked in the office and 2 did business (Table 4).

**Table 4:** Characteristics of 9 PKDL patients.

Characteristics	Number (%)
*District*	
Morang	4(44.4)
Surkhet	4(44.4)
Siraha	1(11.1)
*Municipality*	
Rangeli	4(44.4)
Birendranagar	4(44.4)
Laxmipur	1(11.1)
*Age distribution (years)*	
<20	2(22.2)
20–45	5(55.6)
46–60	1(11.1)
>60	1(11.1)
*Gender distribution*	
Male	4(44.4)
Female	5(55.6)
*Education status*	
No formal education	2(22.2)
Literate	1(11.1)
Primary	2(22.2)
Secondary	4(44.4)
*Occupation*	
Farmer	2(22.2)
Housewife	1(11.1)
Labor	1(11.1)
Business	2(22.2)
Student	2(22.2)
Job in office	1(11.1)

Among the 9 PKDL cases, 4 had macular lesions, 2 had polymorphic lesions and 1 each had papular, papular and nodular, and macular and papular lesions. All those lesions were in the exposed body part of the patients. Among the 9 cases 8 were detected through active case detection and 1 through self-referral.

### Assessment of knowledge of PKDL, perceived stigma related to the disease and treatment seeking behavior of PKDL patients

Among the 9 PKDL cases, 5 had present or previous VL cases in the household. All PKDL cases had heard of VL but only one had heard of PKDL. One PKDL case mentioned skin lesions as a symptom of PKDL and PKDL as a reservoir of VL. The rest did not know the symptoms as well as the role of PKDL in transmission of VL. All of the PKDL patients mentioned that they would get treatment from the government hospitals.

Out of a total of 36 maximal points in the stigma score, the mean score was 17.89±12.84 (min = 1, max = 33). Three had a stigma score of less than 10, 2 had a stigma score of between 10–20 and 2 had stigma score between 21–30 and 2 had a score of greater than 30 (Table 5).

**Table 5:** Stigma score and knowledge of PKDL patients on VL and PKDL.

Particulars	Values
*Mean stigma score*	17.89±12.84(min = 1, max = 33)
*Frequency of stigma score*	
<10	3(33.3)
10–20	2(22.2)
21–30	2(22.2)
>30	2(22.2)
*Heard about kala-azar*	
Yes	9 (100.0)
No	0 (0.0)
*Heard about PKDL*	
Yes	1 (11.1)
No	8 (88.9)
*Knowledge of symptoms of PKDL*	
Skin lesions	1 (11.1)
Fever	1 (11.1)
Don’t know	7 (77.8)
*Know that PKDL is a reservoir of VL*	
Yes	1 (11.1)
No	8 (88.9)
*Vectors for VL*	
Sandfly	2 (22.2)
Mosquito	1 (11.1)
Don’t know	6 (66.7)
*Breeding sites of sandfly*	
Moist and dirty places	5 (55.6)
Don’t know	4 (44.4)

The details of the 12 points stigma scale has been given in S1 Table.

### Integrated approach of detecting PKDL among cases with skin lesions in hospitals with a focus on management of leprosy cases

In two hospitals with a focus on management of leprosy cases, applying the integrated approach of detecting PKDL among cases with skin lesions negative for leprosy, the following was found: 9 cases were tested for PKDL and none of them found positive for PKDL in Lalgadh Leprosy Hospital but no one was tested in Anandaban Leprosy Hospital as there was no one with suspected PKDL. One of the cases was referred to Sukraraj Tropical and Infectious Disease Hospital in Kathmandu but confirmatory tests showed that this was indeed a leprosy case.

### Number of new VL cases that arose from each PKDL case

We also collected data on VL and PKDL cases in the past during screening of PKDL in the villages. It is difficult to ascertain the temporal pattern of PKDL and VL. However, it was found that in Morang there were 4 VL cases close to 4 PKDL cases. In Siraha, 5 VL cases were reported from the same village as 1 PKDL case. In Surkhet, there was 1 PKDL case and 1 VL case in 3 villages and 2 VL cases in the fourth villages with a PKDL case (Table 6).

**Table 6:** VL cases around PKDL cases.

District	Village	Number of PKDL cases	Number of VL cases
Morang	Babiyabirta	3	2
	Dohamana	1	2
Siraha	Rampur	1	5
Surkhet	Narayantol	1	1
	Sathkhola	1	2
	Mulpani	1	1
	Ghariban	1	1

## Discussion

Our study found that the prevalence of PKDL in Nepal was 2.23 per 10,000 population. This burden is lower than that in Bangladesh and India. A follow up of VL treated cases between 2000–2009 in Nepal revealed that 2.4% of them had developed PKDL [5]. A similar study in Bangladesh showed, that the incidence of PKDL in VL patients treated with different anti-VL drugs was 12.3% [3]. The prevalence of PKDL in India was 4.4 per 10,000 population [9]. In contrast, in Nepal a community based survey for detection of VL/PKDL along with other febrile diseases through integrated active case detection in 2015 and 2014 in a limited population did not find any PKDL case [10, 11]. The clustering of PKDL from Surkhet and Morang coming from the same municipality with an overall prevalence of 11.54 in Surkhet and 4.02 in Morang per 10,000 population indicated a much larger prevalence. The clustering of cases from these two districts is likely to be due to the high *L*. *donovani* endemic areas in these districts or could also be due to propensities towards incomplete treatment [12]. Among 147 cases with skin lesion, only 9 were positive for PKDL. All suspected skin manifestations were validated by dermatologists. Beside PKDL other manifestations were mostly diagnosed as scabies and psoriasis. The low number of PKDL cases detected might be due to under diagnosis of PKDL by the dermatologists. Some PKDL cases may have been missed or misdiagnosed as some other skin disease. Therefore, further training of dermatologists on the differential diagnosis of PKDL would be useful for timely treatment. Training of community level health workers on the diagnosis of PKDL would help to achieve timely detection and referral of cases for treatment.

PKDL cases without a past history of VL in districts with new foci made up around half of the total number of PKDL cases encountered. The reasons for such occurrence are still not known. A study conducted in Bihar, India, in 2014–2015 also reported that 23% of the PKDL cases had no prior history of VL [13]. In recent years VL has been significantly reduced in Bangladesh, India and Nepal; recent data on sub-clinical VL cases in Nepal is not available. However, the studies conducted in India in 2007 and 2014–2015 revealed that sub-clinical seropositive VL cases were 15.7% and 2.3% of all cases respectively [14, 13]. Sub-clinical infection of *L*. *donovani* might be a risk factor for developing the PKDL. An association between chronic environmental arsenic exposure and the development of PKDL has also been suggested [13]. Further, research is needed particularly in new VL foci to identify the reasons for PKDL occurrence in cases without a past history of VL.

In our study among PKDL cases with a past history of VL, 3 reported that they had not complete their treatment of VL. Although risk factors for PKDL are not well established, incomplete treatment continues to be understood as one important risk factors for the development of PKDL [6]. One study conducted in Bangladesh revealed that there was a lower incidence of PKDL among cases treated with SSG and multi-dose L-AmB compared to other treatment regimens [3]. In our study due to the small number of PKDL cases, we could not further analyze if a particular drug is a risk factor for PKDL, but it can be seen that current VL drugs could not prevent PKDL development.

Macular skin lesions were frequently found among PKDL cases in Nepal. Such skin lesions are common among PKDL cases in Asia whereas papular lesions are more common in PKDL patients of Africa [15]. A recent study in India showed that 90% of PKDL cases had macular lesions [16]. Macular cases are less likely to seek treatment and may be easily missed. In this study, we presented all kinds of cases with skin lesions to the clinical experts in hospitals. The macular cases of PKDL were more likely to be considered as leprosy. Therefore, integrated screening of skin diseases in the community will detect such cases of PKDL and leprosy. The macular cases of PKDL have a substantial parasite load [17], and they can transmit parasites to sandflies and can play a role in VL transmission [18].

Similar to leprosy, PKDL patients may face stigma which affects their treatment seeking behaviour. We assessed the knowledge of PKDL patients on VL, VL vectors and PKDL as well as stigma level of the PKDL patients. We found that most of them were aware of VL but not of PKDL. They had perceived some level of stigma, some of them even a high level. In Bihar, India, PKDL treatment delays were very long and patients had poor knowledge of VL, PKDL and VL vectors with feelings of stigmatization [19]. Awareness programs on VL and PKDL should be conducted regarding the disease and its linkage with VL thus reducing the stigma among the patients.

We did not find PKDL cases among leprosy negative cases in the hospitals with a focus on management of leprosy cases through the integrated approach. This might be due the low prevalence of PKDL [11, 20]. We still recommend that regular implementation of integrated approach of PKDL detection in active case detection camps and leprosy hospitals will be useful in the consolidation and maintenance phase of VL elimination since a single case of PKDL can potentially cause a new VL outbreak.

We were not able to assess the temporal pattern of PKDL and VL but we found an association as in most villages with PKDL there were also VL cases. One of the studies in India mentioned that PKDL is prevalent in areas where VL is common [21] but another study in India showed that there were no new cases of VL detected in the PKDL houses for upto 18-months of follow-up [22]. Either VL or PKDL cases might be contributing to the transmission of parasites which needs to be ascertained through further studies.

There is no active case search for PKDL in the national programme to date. Only passively reported PKDL cases have been included in the programme. But as PKDL cases are potential reservoirs of *L*. *donovani* parasites, early detection, diagnosis and treatment of cases is essential to maintain the elimination status of VL.

In conclusion, the burden of PKDL is low in both previously VL endemic and new focal areas of Nepal. Currently used VL treatment regimens could not prevent the development of PKDL. There is very limited knowledge of PKDL and VL among PKDL cases and they had perceived stigma. Continuous surveillance is essential in the maintenance phase of VL elimination since PKDL can be a reservoir for the transmission of VL. Awareness raising activities on VL and PKDL should be conducted in VL endemic areas to promote knowledge and reduce stigma in the society. Integrated approach of detecting PKDL in hospitals with a focus on management of leprosy cases should be continued. Further research is needed in new VL foci to identify the reasons for PKDL occurrence in cases without past history of VL.

## Supporting information

S1 Table Stigma scale of the PKDL patients. (DOCX)
