# Supplementary material for: Prevalence of post kala-azar dermal leishmaniasis (PKDL) and treatment seeking behavior of PKDL patients in Nepal
Source: PLoS Negl Trop Dis. 2023 Feb 9;17(2):e0011138. doi: 10.1371/journal.pntd.0011138 (PMC9946221; doi:10.1371/journal.pntd.0011138)
Supplement: S1 Table — (DOCX) [file pntd.0011138.s001.docx]

**Supplementary information**

Table S1: Stigma scale of the PKDL patients

| **Items** | **No-0** | **Uncertain-1** | **Possibly-2** | **Yes-3** |
| --- | --- | --- | --- | --- |
| Should let others know | 4(44.4) | 1(11.1) | 1(11.1) | 3(33.3) |
| Think less of yourself | 6(66.7) | 1(11.1) | 0(0) | 2(22.2) |
| Embarrassed or shamed | 4(44.4) | 2(22.2) | 0(0) | 3(33.3) |
| Receive less respect from other | 5(55.6) | 0(0) | 1(11.1) | 3(33.3) |
| Adverse effect on others | 2(22.2) | 1(11.1) | 2(22.2) | 4(44.4) |
| Others avoided you | 4(44.4) | 1(11.1) | 0(0) | 4(44.4) |
| Refuse to visit your home | 4(44.4) | 1(11.1) | 0(0) | 4(44.4) |
| Others think less of your family | 3(33.3) | 0(0) | 1(11.1) | 5(55.6) |
| Difficult to marry (if unmarried) | 3(33.3) | 1(11.1) | 0(0) | 5(55.6) |
| Problems in family (if married) | 4(44.4) | 0(0) | 1(11.1) | 4(44.4) |
| Asked to stay away from work | 4(44.4) | 1(11.1) | 0(0) | 4(44.4) |
| Decided to stay away from social groups | 2(22.2) | 1(11.1) | 2(22.2) | 4(44.4) |
